# Seroprevalence of human adenovirus type 5 neutralizing antibodies in the Philippines

**DOI:** 10.1371/journal.pone.0293046

**Published:** 2023-12-01

**Authors:** Abialbon G. Francisco, John Carlo B. Reyes, Ian Kim B. Tabios, Criselda Jean G. Cruz, Mark Angelo C. Ang, Francisco M. Heralde, Azita Racquel G. Lacuna, Sheriah Laine M. de Paz-Silava

**Affiliations:** 1 Department of Medical Microbiology, College of Public Health, University of the Philippines Manila, Manila, Philippines; 2 Department of Laboratories, Philippine General Hospital, University of the Philippines Manila, Manila, Philippines; 3 Institute of Biology, College of Science, University of the Philippines Diliman, Quezon City, Philippines; 4 Department of Biochemistry and Molecular Biology, College of Medicine, University of the Philippines Manila, Manila, Philippines; 5 Department of Dermatology, National Cheng Kung University Hospital, College of Medicine, National Cheng Kung University, Tainan, Taiwan; 6 International Center for Wound Repair and Regeneration, National Cheng Kung University, Tainan, Taiwan; 7 College of Medicine, University of the Philippines Manila, Manila, Philippines; 8 Department of Pathology, College of Medicine, University of the Philippines Manila, Manila, Philippines; University of Georgia, UNITED STATES

## Abstract

Human adenovirus (HAdV), particularly the HAdV type 5 (HAdV-5), has been extensively utilized in the development of vector vaccines due to its high immunogenicity, good safety profile, and ease of propagation. However, one of the main challenges in its use is the presence of pre-existing immunity among vaccine recipients. Pre-existing neutralizing antibodies (NAbs) can prevent the uptake of HAdV-5 vectors and reduce vaccine efficacy. Hence, this study investigated the seroprevalence of NAbs against HAdV-5 in urban and rural regions of the Philippines. Luciferase-based neutralization assay was performed on 391 plasma/serum samples. Out of these samples, 346 or 88.5% were positive for HAdV-5 NAbs, and the majority of them (56.8%) had high titers against the virus. Among the regions included in this study, Bicol (Region V) had the highest seroprevalence rate (94.1%). Our findings show that a significant number of adults in the Philippines have pre-existing immunity against HAdV-5. This supports the recommendation that vaccination programs in the country should consider implementing vaccination techniques, such as a prime-boost regimen or addition of booster doses, to address the potential negative effects of pre-existing HAdV-5 immunity in the efficacy of adenoviral vector-based vaccines.

## Introduction

Human adenoviruses (HAdVs) are non-enveloped, icosahedral viruses with double-stranded DNA genome [1]. They are composed of seven subgroups (A-G) and more than 85 serotypes [2]. Human transmission occurs via respiratory droplets, fecal-oral route, and contact through fomites [3]. Most infections are self-limiting but usual clinical signs and symptoms include diarrhea, gastroenteritis, cough, common colds, sore throat, pharyngitis, tonsillitis, fever, and keratoconjunctivitis [1,4,5]. Outbreaks of infections are usually reported in crowded settings like in hospitals, nursing homes, schools, and military bases [6–8].

For the past decades, HAdV vectors have been extensively studied for their applications in gene therapy, cancer therapy, and vaccine design due to several advantages. They can infect both non-dividing and dividing cells, they are easy to cultivate and propagate into high titers, they are highly immunogenic, and they are deemed safe due to their non-integration into the host’s genome [9–11]. HAdV-5 is usually the preferred vector due to its higher immunogenicity compared to other HAdV serotypes [12]. Numerous human clinical trials of adenovirus vector-based vaccines have been conducted against influenza virus, Ebola virus, *Mycobacterium tuberculosis*, and human immunodeficiency virus (HIV) [13–17]. Also, this vaccine platform has been recently used against severe acute respiratory syndrome coronavirus 2 (SARS-CoV-2) which causes coronavirus disease-2019 (COVID-19) [18,19]. Most clinical trials have shown its good safety profile and tolerability [11].

A major challenge for the use of this vaccine type is the presence of pre-existing NAbs against the adenovirus vector in the general population. It is believed that more than 80% of the human population has been naturally exposed to at least one serotype of HAdV especially during childhood [20–22]. Pre-existing antibodies neutralize the hexon, penton, and fiber proteins of the adenovirus capsid, which greatly reduce the uptake of the vectors and expression of its transgene [23,24]. These effects of pre-existing antibodies on HAdV-5 vectors have been reported in several studies involving mice, rhesus monkeys, and humans [25–27].

Various studies have been conducted to assess the seroprevalence of NAbs against HAdV-5, but there is limited published data in the Philippines which utilized several adenoviral-based vaccines in its COVID-19 vaccination program. Last April 2023, a total of 181.6 million doses of COVID-19 vaccines were administered in the population [28]. Out of these vaccine doses, AstraZeneca’s ChAdOx1-S had the largest share with 23.9 million doses (13.2%), followed by Janssen’s Ad26.COV2-S with 7.7 million doses (4.2%), and Gamaleya Institute of Russia’s Sputnik V/Sputnik Light with 1.6 million doses (0.9%). Given the known effects of NAbs with the efficacy of adenovirus vector-based vaccines, it is of paramount importance to determine the seroprevalence of HAdVs. The findings of the study may provide valuable insights into the future development of these vector vaccines and its administration in the local population. In particular, it can be used to assess the appropriate strategy in order to circumvent the potential negative effects of NAbs against these vaccines. Hence, the present study determined the seroprevalence of HAdV-5 NAbs in the Philippine general population using biobanked plasma/serum samples.

## Materials and methods

### Study design

This study utilized an analytical cross-sectional study design. Plasma samples were sourced from plasma units discarded by the Blood Bank Division of the University of the Philippines–Philippine General Hospital (UP-PGH) due to storage limitations, while biobanked serum samples were retrieved from the Institute of Biology, University of the Philippines Diliman (SJREB-2019-37; UPMREB 2021-0569-01) from February to October 2021.

### Eligibility criteria

#### Inclusion criteria

The following are the inclusion criteria of the study:

- Fresh frozen plasma (FFP) samples from volunteer non-remunerated Filipino adults who met the standard of the Philippine Department of Health, World Health Organization, and Association for the Advancement of Blood and Biotherapies eligibility criteria for blood donation;
- Completely filled-out blood donor forms: (i) UP-PGH Blood Donor History Questionnaire, (ii) Physical Examination Form, and (iii) Informed Consent for Donation;
- Serum samples with informed consent/assent forms and complete demographic information.

#### Exclusion criteria

The following are the exclusion criteria of the study:

- Insufficient volume of aliquot sample (<100 μl) for virus neutralization assay;
- Expired blood products (units beyond the recommended 12-month shelf life of the Philippine Department of Health, World Health Organization, and Association for the Advancement of Blood and Biotherapies);
- Units with evidence of freeze-thaw cycling;
- Units with evidence of bacterial growth or contamination.

### Sample size

The minimum sample size required to estimate the seroprevalence of HAdV-5 neutralizing antibodies among Filipino blood donors was calculated using OpenEpi online program version 3.0 (http://openepi.com/SampleSize/SS Propor.htm/). A minimum of 289 blood donor samples was needed to estimate a 75% seroprevalence of adenovirus neutralizing antibodies, with a margin of error of ±5 percentage points and 95% confidence level. A total of 391 plasma/serum samples were analyzed for the presence of HAdV-5 neutralizing antibodies. Random sampling was performed using a pseudorandom number-generating function in Google Sheets. Sampling was performed until the set of sample size was reached.

### Virus and host cell cultivation

Premade E1/E3-deleted, replication-incompetent recombinant adenovirus type 5 with cytomegalovirus (CMV) promoter expressing firefly luciferase and green fluorescent protein (rAd5-Luc/GFP) (Amsbio, United Kingdom) were used. Human embryonic kidney (HEK) 293T cells (ATCC, USA) were grown as monolayers for viral propagation on tissue culture T-75 flasks and maintained in complete growth medium containing Dulbecco’s Modified Eagle’s Medium (DMEM) (Gibco, USA) supplemented with 10% heat-inactivated fetal bovine serum (FBS) (Gibco, USA), and 1% penicillin-streptomycin and amphotericin B (Gibco, USA) in 5% CO_2_ humidified atmosphere at 37°C. Cell viability and density were regularly monitored by trypan blue staining.

### Viral propagation and purification

The stock of rAd5-Luc/GFP was propagated and purified following the procedures of the commercial kit, Adeno-X Maxi Purification Kit (Clontech Laboratories Inc., USA). The cells were infected with various dilutions of the viral stock, starting from multiplicity of infection (MOI) of 4, 2, 1, and 0.5. They were incubated for four days at 37°C and 5% CO₂ until the cytopathic effect (CPE) was completed. The viral MOI which produced the 50% CPE was the optimum amount of virus for the next purification steps of rAd5-Luc/GFP. HEK-293T cells were pooled into a 50-ml conical tube with a final density of 6.92 x 10⁶ cells/ml. The cells were infected with the optimal amount of viral MOI and incubated for several days. The propagated rAd5-Luc/GFP was harvested by centrifugation of the virus-infected cells, and the resulting pellet was lysed by several freeze-thaw cycles. The lysate was filtered and further purified using the pre-assembled purification filter.

### Viral titration

The titer of the purified rAd5-Luc/GFP was measured following the protocol of Adeno-X Rapid Titer Kit (Clontech Laboratories Inc., USA). HEK-293T cells were seeded onto a 24-well plate. Ten-fold serial dilution of rAd5-Luc/GFP was performed and added to each well of the plate. After incubation, the cells were fixed using ice-cold 100% methanol. The cells were washed several times and the anti-hexon antibody was added to each well. It was followed by the addition of a horseradish peroxidase-conjugated anti-mouse antibody. After washing, a diaminobenzidine (DAB) working solution was finally added to the wells of the plate. The virus dilution which produced the ideal number of positive cells in the plate was used for the computation of the viral titer following manufacturer’s instructions.

### Viral neutralization test by luciferase assay

HAdV-5 neutralizing antibodies were quantified by luminescence-reduction neutralization tests (Promega, USA) and as described in [29]. Assays were optimized prior by determining the optimal viral MOI. Plasma/serum samples were heat inactivated at 56°C for 1 hour before serial dilution in a 96-well plate. Four-fold serial dilution of samples was performed starting from 1:16 to 1:16,384 to achieve a final volume of 12.5 μl. In another 96-well white plate, 12.5 μl of serially diluted samples were dispensed to each well. Next, 12.5 μl of virus solution (MOI 0.5) was added to each well followed by the addition of 25 μl of HEK-293T cells (1.0x10^4^ cells/well). Plates were incubated in 5% CO_2_ at 37°C for 24 hours. Then, 50 μl of ONE-Glo luciferase reagent was added to each well and the plate was analyzed using the Promega GM3500 multimode microplate reader. Background luminescence was normalized with wells containing only HEK-293T cells, while maximum luminescence was provided by the negative control wells containing cells infected with rAd5-Luc/GFP. Each plasma/serum sample was tested in duplicate.

Neutralizing titer refers to the reciprocal of plasma/serum dilution which produced at least 50% reduction in luminescence as compared to the negative control [28]. Titers <16 were considered negative for the presence of neutralizing antibodies while titers ≥16 were reported as positive for NAbs. The measured HAdV-5 NAb titers were also classified into two categories of either low (≤200) or high titer (>200) which are based on recent clinical trials that evaluated the effects of pre-existing NAbs against HAdV-5 vector vaccines [19,30–32].

### Statistical analysis

**Fig 1:**
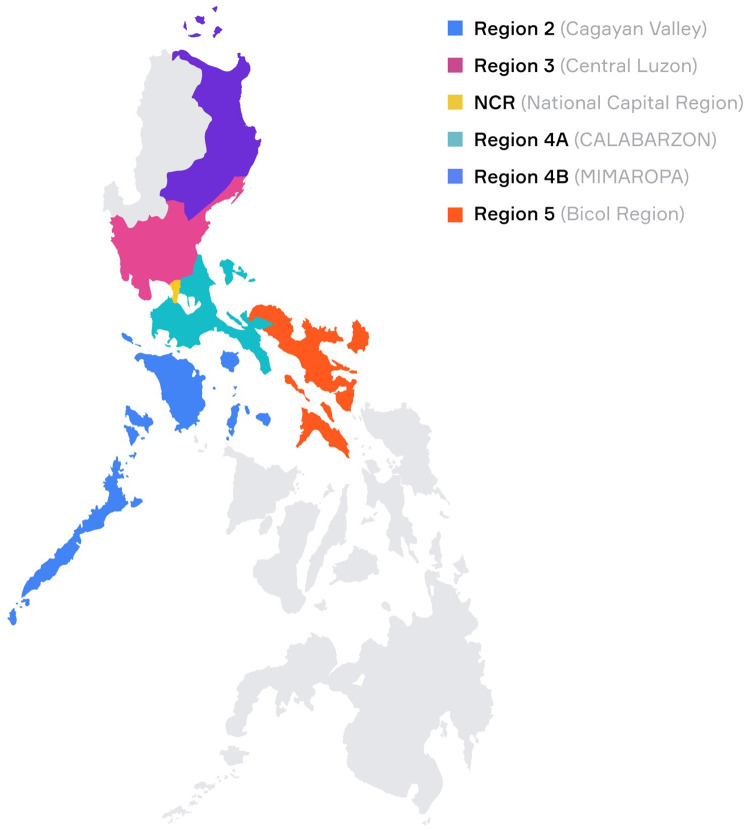
Map of the Philippines showing the residence of blood donors. Highlighted regions indicate the place of residence of blood donors.

The raw data were encoded into Microsoft Excel and loaded into IBM SPSS version 29. Demographic data and proportions were summarized into graphs using the software GraphPad Prism 10. Age in years was binned into six age groups and summarized in counts and percentages of total subjects. Sex was summarized in counts and percentages. Blood donors came from six different regions of the country as shown in **Fig 1**. Due to the low number of donors and the proximity of these regions to each other, the counts of Regions II (Cagayan Valley) and III (Central Luzon), and Regions IV-A (CALABARZON) and IV-B (MIMAROPA) were combined in the analysis. Region of residence was categorized into four main groups: 1) National Capital Region (NCR), 2) Region II and Region III, 3) Region IV-A and Region IV-B, and 4) Region V (Bicol Region), and summarized in counts and percentages. In a 2021 report of the Philippine Statistics Authority, NCR had the lowest poverty incidence rate among families (2.2%) followed by Regions IV-A and III with 7.2% and 8.3%, respectively [33]. On the other hand, Region V had the highest poverty incidence among the regions (21.9%). Region II had a poverty incidence of 11.7% while Region IV-B had 15%. The seroprevalence of NAbs against HAdV-5 was estimated with 95% level of confidence. The levels of neutralizing antibodies against HAdV-5 were summarized using the geometric mean and geometric standard deviation. The NAb titers were natural-log transformed before statistical analysis. One-way ANOVA and t-test for independent groups were used to test the hypothesis of no difference in order to compare the geometric mean titer (GMT) values among subgroups of age and residence, and males and females, respectively. Odds ratios or coefficient effects with *p*-values <0.05 were considered statistically significant.

### Ethical considerations

The study was conducted in compliance with the Philippine Republic Act 10173 (Data Privacy Act of 2012). Permission to utilize the discarded plasma samples was obtained from the University of the Philippines Manila Research Ethics Board (UPMREB 2020-0726-01). The anonymized sociodemographic information of the donors was retrieved from the data managers and accessed on 29 April 2022. Permission to utilize the biobanked serum samples was secured from Single Joint Research Ethics Board of the Philippine Department of Health (SJREB 2019–37) and UPMREB (UPM-REB 2021-0569-01). The anonymized sociodemographic information of the donors was retrieved from the data managers and accessed on 27 September 2022. No identifiers or sensitive information were collected from the blood donors. Plasma and serum samples with written informed consent were included and given corresponding codes in order to ensure the anonymity of the subjects.

## Results

### Optimization of the luciferase assay

**Fig 2:**
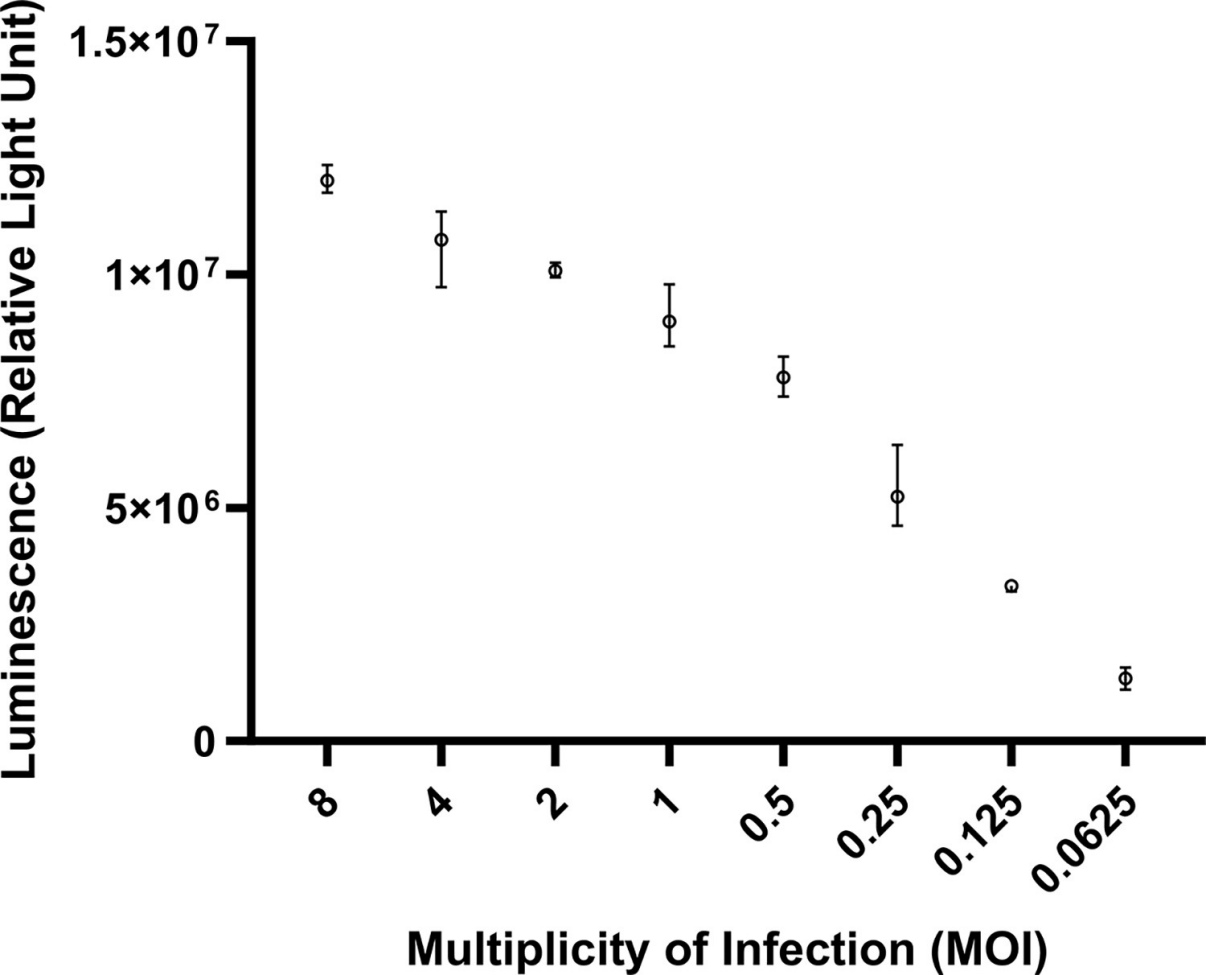
Luminescence values of different MOIs of rAd5-Luc/GFP. Each dot indicates the mean luminescence value while minimum and maximum luminescence values are represented as whiskers in the graph. Each viral MOI was tested in triplicates.

Several MOIs of rAd5-Luc/GFP were tested in order to determine the optimal titer for the luciferase assay. **Fig 2** shows that there was a direct relationship between luminescence and multiplicity of infection (MOI) such that as the viral MOI decreases starting with MOI 8, the luminescence value or relative light unit (RLU) also decreases. However, three of the highest viral MOIs (MOI of 64, 32 and 16) exhibited lowest RLU values possibly due to cell death and toxicity. MOI of 0.5 produced a high luminescence value that was located in the middle of the linear curve. Hence, it was selected to be used for the luciferase assay of the samples.

### Demographic characteristics of the study population

A total of 391 plasma/serum samples were collected and tested as described above. The age of the donors ranged from 13–75 years old with a mean of 34.2 years old. About 40.9% of the plasma/serum donors belonged to the age group of 18–29 years old (**Table 1**). Majority of the donors were males (71.1%) and most of them were residing in the National Capital Region (44.5%) followed by Regions IV-A and IV-B (26.6%).

**Table 1:** Demographic characteristics of plasma/serum donors of the study (N = 391).

Characteristics		n (%)
**Age (in years)**	<18	9 (2.3)
	18–29	160 (40.9)
	30–39	107 (27.4)
	40–49	53 (13.6)
	50–59	47 (12)
	60–79	15 (3.8)
**Sex**	Male	278 (71.1)
	Female	113 (28.9)
**Residence**	National Capital Region	174 (44.5)
	Regions II and III	11 (2.8)
	Regions IV-A and IV-B	104 (26.6)
	Region V	102 (26.1)

### Seroprevalence of human adenovirus 5 neutralizing antibodies in the study population

**Fig 3:**
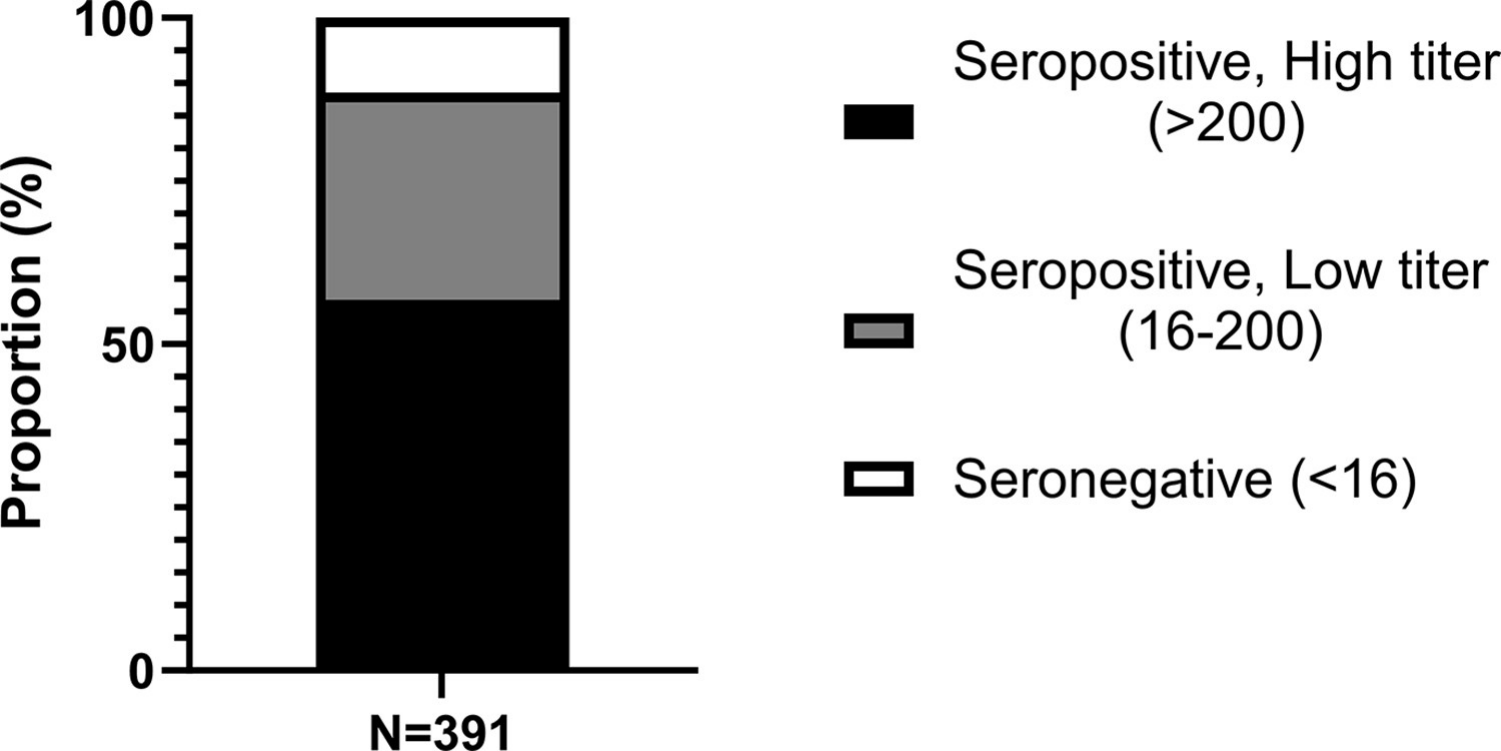
Distribution of HAdV-5 neutralizing antibody titers among donors of plasma/serum (N = 391). Black bar represents the seropositive high titer donors (>200). Gray bar shows the seropositive low titer donors (16–200). White bar depicts seronegative donors (<16).

Luciferase-based neutralization assay was performed in order to determine the presence of HAdV-5 NAbs among the collected plasma/serum samples. Out of 391 samples, 346 or 88.5% (95% confidence interval: 85–91.3%) were positive for HAdV-5 NAbs. Among the seropositive samples, 222 donors (56.8%) had high titers against HAdV-5 while 124 donors (31.7%) had low titers. **Fig 3** shows the distribution of HAdV-5 NAb titers in the study population.

### Distribution of HAdV-5 seropositivity according to age groups

**Fig 4:**
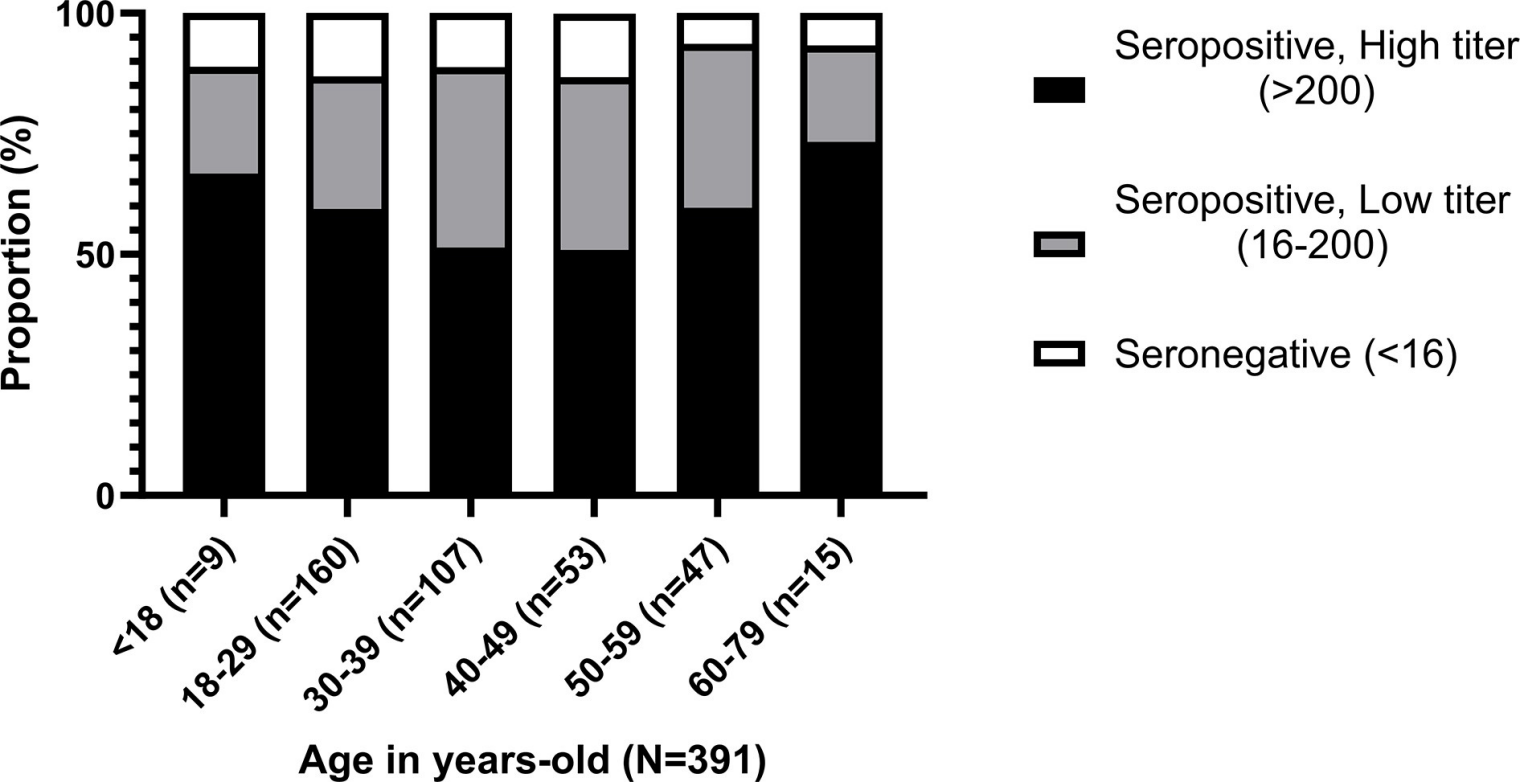
Distribution of HAdV-5 seropositivity according to age groups (N = 391). Black bar represents the seropositive high titer donors (>200). Gray bar shows the seropositive low titer donors (16–200). White bar depicts seronegative donors (<16).

The seroprevalence rate of HAdV-5 NAb in age groups of <18 years old, 18–29 years old, 30–39 years old, 40–49 years old, 50–59 years old, and 60–79 years old are 88.9% (95% confidence interval: 56.5–98%), 86.9% (95% confidence interval: 80.8–91.3%), 88.8% (95% confidence interval: 81.4–93.5%), 86.8% (95% confidence interval: 75.2–93.5%), 93.6% (95% confidence interval: 82.8–97.8%), and 93.3% (95% confidence interval: 70.2–98.8%), respectively. A trend of higher seroprevalence was observed in younger and older donors with no significant difference among their proportions (p = 0.863, Fisher’s exact test). The age group of 60–79 years old had the highest proportion of high seropositive donors (73.3%) followed by donors <18 years old (66.7%). The age groups of 50–59 years old, 18–29 years old, 30–39 years old and 40–49 years old had 59.6%, 59.4%, 51.4% and 50.9% of high seropositive donors, respectively. **Fig 4** shows the distribution of HAdV-5 NAb titers in each age group.

### Distribution of HAdV-5 seropositivity according to sex

**Fig 5:**
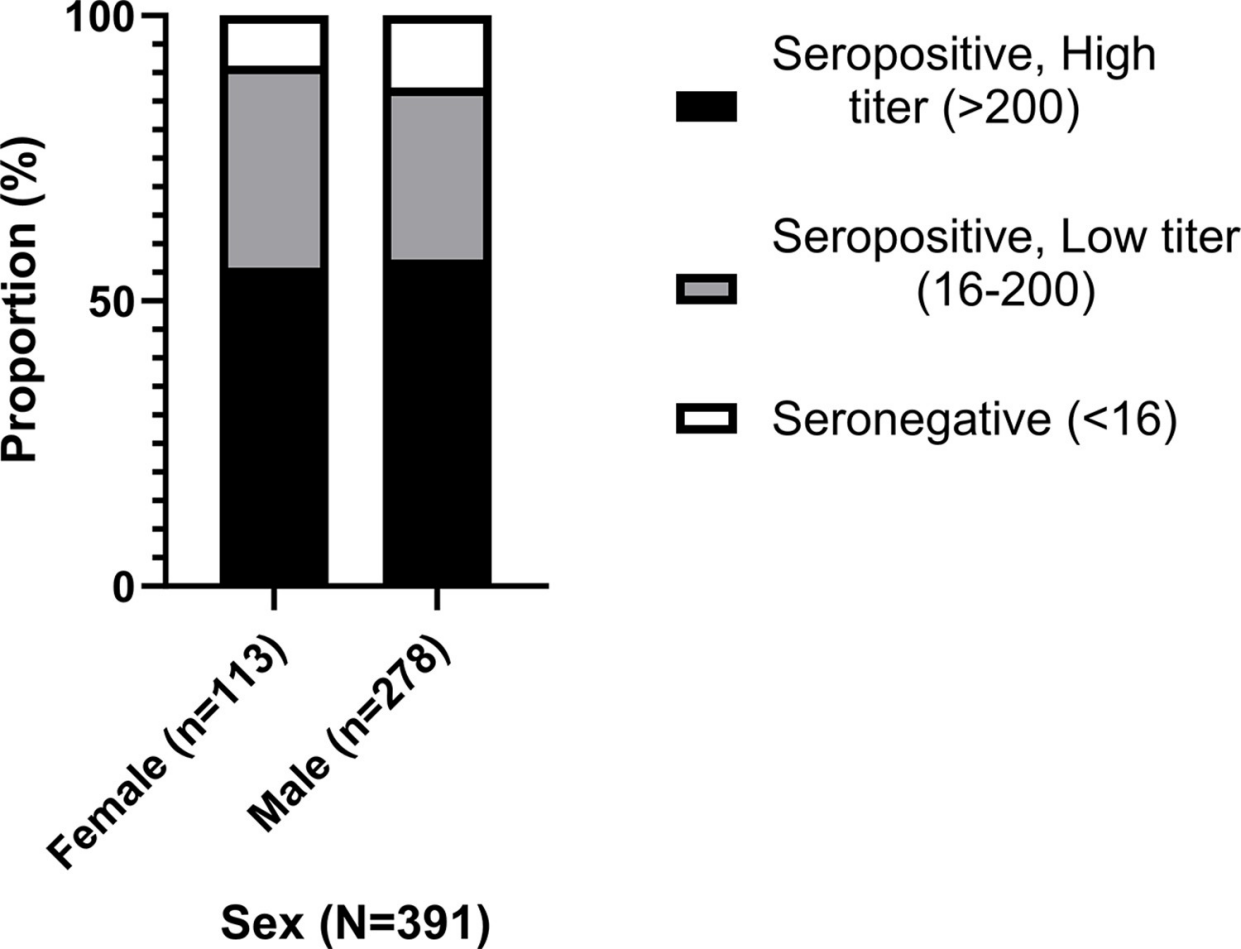
Distribution of HAdV-5 seropositivity according to sex (N = 391). Black bar represents the seropositive high titer donors (>200). Gray bar shows the seropositive low titer donors (16–200). White bar depicts seronegative donors (<16).

Females showed a trend towards a higher seroprevalence of 91.2% (95% confidence interval: 84.5–95.1%) compared to males with 87.4% (95% confidence interval: 83–90.8%) with no significant difference between them (p = 0.293, z-test of independent proportions). Male donors had higher proportion of high seropositive donors (57.2%) than female donors (55.8%). **Fig 5** depicts the distribution of HAdV-5 NAb titers according to sex.

### Distribution of HAdV-5 seropositivity according to residence

**Fig 6:**
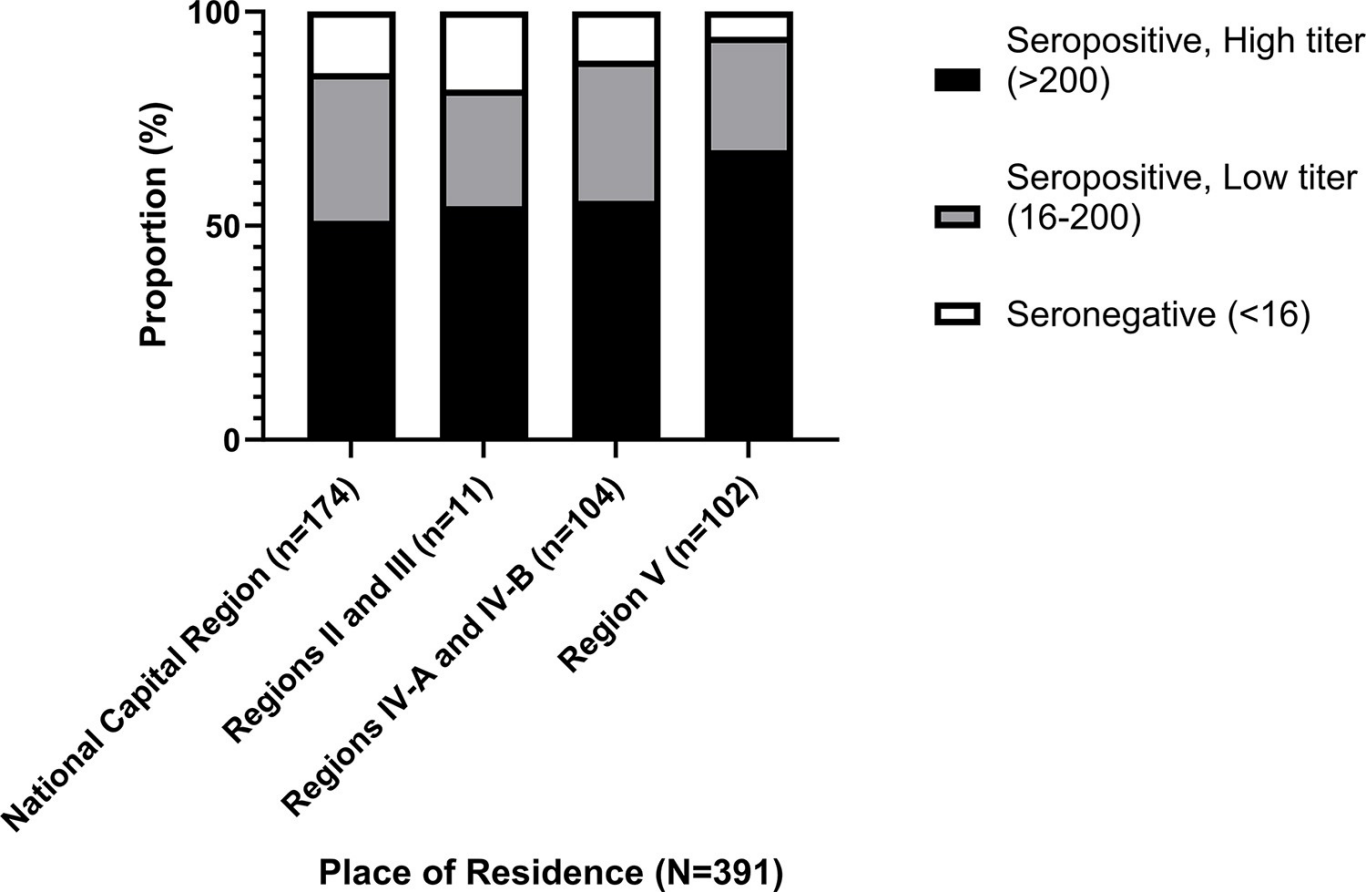
Distribution of HAdV-5 seropositivity according to residence (N = 391). Black bar represents the seropositive high titer donors (>200). Gray bar shows the seropositive low titer donors (16–200). White bar depicts seronegative donors (<16).

The seroprevalence rates of HAdV-5 NAb in the National Capital Region, Regions II and III, Regions IV-A and IV-B, and Region V are 85.6% (95% confidence interval: 79.7–90.1), 81.8% (95% confidence interval: 52.3–94.9%), 88.5% (95% confidence interval: 80.9–93.3%), and 94.1% (95% confidence interval: 87.8–97.3%), respectively. A trend of higher seroprevalence was observed in rural regions compared with urban region (NCR). However, no significant difference was found among their proportions (p = 0.119, Fisher’s exact test). Donors coming from Region V had the highest proportion of high seropositivity (67.6%) followed by Regions IV-A and IV-B (55.8%). Donors from Regions II and III, and NCR had 54.5% and 51.1% of high seropositivity, respectively. **Fig 6** shows the distribution of HAdV-5 NAb titers based on place of residence.

### Geometric mean titers of HAdV-5 NAb of the study population

The geometric mean NAbs titers were calculated for each level of the demographic factors. (**Table 2**). The overall NAbs GMT of the study population is 167.2. The age group of <18 yrs. old had the highest GMT (254.2) followed by age groups 60–79 (232.4) and 50–59 (201.4). Females had higher GMT (194.3) than males (157.3). These differences were however not found to be statistically significant. In terms of residence, donors who came from or resided in Region V had the highest NAbs GMT (280.5) followed by donors who came from Regions IV-A and IV-B (159.4). In order to compare the GMT values of each demographic factor, they were naturally log-transformed and their arithmetic means were computed. One-way ANOVA and t-test of independent samples were performed in order to compare the natural log titer of subgroups of age and residence, and males and females, respectively. We found that donors from Region V had statistically higher NAbs titers compared to donors from NCR (p value = 0.01).

**Table 2:** GMT of HAdV-5 NAbs according to demographic factors.

Characteristics	N	GMT	Mean of log titer (Lnª)	p-value	SD^d^	Median (IQR)^e^
**Overall**	391	167.2	5.12	-	1.77	5.55 (2.77)
**Age (in years)**						
<18	9	254.2	5.54	0.56^b^	1.97	5.55 (3.47)
18–29	160	177.9	5.18		1.78	5.55 (2.77)
30–39	107	129.6	4.86		1.63	5.55 (1.39)
40–49	53	167	5.12		1.79	5.55 (2.77)
50–59	47	201.4	5.31		2.08	5.55 (1.39)
60–79	15	232.4	5.45		1.44	5.55 (2.77)
**Sex**						
Male	278	157.3	5.06	0.12^c^	1.71	5.55 (1.39)
Female	113	194.3	5.27		1.90	5.55 (2.77)
**Residence**						
National Capital Region	174	130.9	4.87	0.01^b^	1.66	5.55 (1.39)
Regions II and III	11	104.7	4.65		1.14	5.55 (1.39)
Regions IV-A and IV-B	104	159.4	5.07		1.73	5.55 (2.77)
Region V	102	280.5	5.64		1.94	5.55 (2.77)

ªNatural logarithm.

^b^One-way analysis of variance among natural log titers.

^c^T-test of independent samples between natural log titers.

^d^Standard deviation of natural log titers.

^e^Median and interquartile range of natural log titers.

### Measuring association between demographic factors and HAdV-5 titers

Two binary logistic regression analyses were done to measure possible associations between demographic factors (age, sex and residence) and the outcomes, HAdV-5 seropositivity and having high HAdV-5 NAb titers (**Table 3**). No demographic factor was observed to be statistically associated with both outcomes (p >0.05).

**Table 3:** Binary logistic regression analysis between demographic factors and two outcomes, HAdV-5 seropositivity and having high titers against HAdV-5 NAb (N = 391).

Demographic factor	n	HAdV-5 Seropositivity		High titer against HAdV-5	
		Odds-ratio	p-valueª	Odds-ratio	p-valueª
**Age (in years)**					
<18	9	Reference level	-	Reference level	-
18–29	160	2.04	0.83	1.36	1.24
30–39	107	2.62	0.97	1.02	0.98
40–49	53	1.55	0.81	0.78	0.98
50–59	47	2.88	1.80	0.96	1.08
60–79	15	2.74	0.92	1.64	1.35
**Sex**					
Female	113	Reference level	-	Reference level	-
Male	278	0.84	0.85	1.26	1.05
**Residence**					
National Capital Region	174	Reference level	-	Reference level	-
Regions II and III	11	0.77	0.75	1.13	1.49
Regions IV-A and IV-B	104	1.36	1.26	1.20	1.10
Region V	102	2.82	0.63	2.36	0.09

ªp-values were adjusted using the Benjamini-Hochberg test.

## Discussion

One of the common vaccine platforms being utilized during the COVID-19 pandemic is viral vectors, particularly those which are derived from HAdV-5 due to its inherent high immunogenicity, good safety profile, high infectivity in different cell types, and ease of propagation [34]. However, high titers of pre-existing NAbs against HAdVs can reduce the immunogenicity and efficacy of these vector vaccines. Hence, it is important to determine the level of NAbs against HAdV-5 in the population.

To date, there is no published study on the seroprevalence of HAdV-5 NAbs in the Philippines. We performed luciferase-based neutralization assay on plasma/serum samples collected from donors in a tertiary hospital and rural province in the country. Out of the 391 samples, 346 or 88.5% (95% confidence interval: 85–91.3%) were positive for the presence of NAbs against HAdV-5. This proportion of HAdV-5 seropositive individuals is comparable to the findings in other low to middle-income countries (LMICs) in the tropics. This may be attributed to several geographic factors such as population density and climate [35,36]. It has been reported that overcrowding contributes to the increased transmission of adenovirus in tropical regions [35]. A study in China suggested that HAdV transmission occurs more easily in tropical regions [36]. Another study reported that military recruits who were exposed to tropical regions for longer periods had high titers against HAdV-4 [37]. Further studies are needed in order to explain the possible roles of these factors.

There is a high seroprevalence rate of HAdV-5 NAbs across different age groups, showing no significant difference in their proportions (p = 0.863). This finding is similar with other studies which reported that HAdV infections occur early in life [35,38]. However, unlike with those studies, the present study did not observe the lowering of proportion of HAdV-5 seropositive individuals as the age increases. It may be due to the change of social behavior being able to move outdoors and increase their exposure to the virus [39]. A study from China suggested that infection rate in adults might be saturated above 18 years old [40]. The present data show that individuals with age ≥13 years have high titers against HAdV-5. Therefore, there is a need to identify a particular age group that will have lower seroprevalence against HAdV-5. A study in Liberia detected the seroprevalence of HAdV-5 among serum samples from infants and the pediatric population (6 months to 18 years old) [41]. They found out that there was a 93% seroprevalence at birth due to maternal antibodies which diminishes after six months of age. Then, it immediately reaches the adult level of HAdV-5 seroprevalence at the age of two. Based on this result, they suggested that age ranging from 6 months to 2 years old can be targeted for vaccine administration in order to avoid the effects of HAdV-5 pre-existing immunity. As for the sex of the serum donors, there was no statistical difference between their seroprevalence rates (p = 0.293). The same finding was reported by other studies conducted in China [36,40,42].

The geometric mean NAb titer of the study population is 167.2, which is lower than that of Thailand population with 669 and Indian population with >1,000 [43,44]. Donors coming from Region V were statistically found to have higher NAb titers against the virus compared to those coming from NCR (p = 0.01). This may be attributed to sanitary conditions of the regions which can also influence the seroprevalence of HAdV-5. Children from villages of China had higher titers of HAdV-5 than the children from the city [45]. People from villages/rural areas are most likely to have less access to clean water and food than people from the city.

The present study detected that the majority of the sample population at 56.8% (95% confidence interval: 51.8–61.6%) had high NAb titers (>200) against the virus. Several studies reported the effects of pre-existing HAdV-5 immunity on HAdV-5 vector-based COVID-19 vaccines. CanSino Biologicals Inc. developed a HAdV-5 vector vaccine against COVID-19 and tested its safety and immunogenicity in a series of clinical trials. At baseline of Phase 1, it measured the level of pre-existing HAdV-5 immunity among participants and divided them into groups of either low (≤200) or high (>200) titers [19]. Those participants who had high titers against HAdV-5 had lower seroconversion rates (25–63%) in all doses after 28 days of vaccination than those participants with low titers (65–85%). In its Phase 2 clinical trial, participants with low titers had seroconversion rates which were twice higher than that of participants with high titers [32]. The same company also tested the immunogenicity of the vector vaccine administered through aerosol inhalation [31]. Similarly, those participants with high pre-existing HAdV-5 immunity had low production of NAbs after vaccination. Furthermore, the vector vaccine was also utilized as a heterologous booster with two doses of inactivated COVID-19 vaccine (Sinovac CoronaVac) administered through oral inhalation [30]. Participants with low pre-existing HAdV-5 immunity had significantly higher antibody responses against the aerosol vaccine.

Numerous approaches have been developed in order to circumvent these effects of HAdV vectors. The first method is to use vectors derived from human adenoviruses with low seroprevalence such as HAdV-35 and HAdV-26 but these vectors have lower immunogenicity and efficacy as compared to that of HAdV-5 [12,46,47]. Vectors derived from non-human species such as chimpanzee adenovirus (ChAdVs) serotype 68 can also be utilized for this purpose [47]. ChAdV-68 binds to the chimeric antigen receptors (CARs) and generates responses similar to HAdV-5 [48]. They have been efficacious in pre-clinical studies but they cross-react with reactive HAdV-specific T-cells due to the close phylogeny between HAdVs and ChAdVs [12,49]. Moreover, changing the administration route of vector vaccines is being employed in order to avoid the recognition by tissue resident memory T-cells [12]. Intranasal booster administration after intramuscular injection can induce memory B and T cells providing mucosal immunity and longer IgG response [47]. Prime-boost immunization is another technique wherein two types of HAdV vectors are being used as prime and booster doses [12]. They can be of identical vector types (homologous) or entirely different (heterologous) such as the Sputnik V or Gam-COVID-Vac of the Gamaleya Institute of Russia. Also, increasing the number of vaccine doses after 3–6 months can also be done [32]. Both immune responses generated by the two latter techniques are sufficient in order to confer protection despite the effects of pre-existing immunity [12,32].

The present study has several limitations. The blood samples were only sourced from six regions of the country, hence, its generalizability is limited only among donors from those regions. Moreover, since we used biobanked sera/plasma, we do not have information on the history of vaccination with an adenovirus-based SARS-CoV-2 vaccine, the roll out of which happened along the same time period of our sample collection. Also, cellular immunity induced by T-cells was not measured in the present study. We recommend exploring HAdV-5 seroprevalence in other regions of the country. Since the seroprevalence of HAdV-5 was detected at a high level, it will be imperative to determine the seroprevalence of other HAdVs (e.g., HAdV-35 and HAdV-26) and those from other sources such as chimpanzee adenovirus. This might identify alternative sources of adenovirus vector for vaccine development. Moreover, it will be important to assess the level of cellular immunity in the population and its possible impact on the efficacy of vector vaccines. Another aspect to explore is the evaluation of the changes in HAdV seroprevalence and NAb titers over time in a certain study population and site.

As of August 2023, the country has recorded more than 4.11 million cases of COVID-19 with over 66,000 deaths [50]. In order to curb the transmission of the disease and its mortality, the government launched its vaccination campaign in March 2021. Since then, there has been an increase in the utilization of adenovirus vector-based COVID-19 vaccines in the country. Our findings suggest that vaccination programs in the country should consider the implementation of vaccination techniques, such as a prime-boost regimen or addition of booster doses, to circumvent potential negative effects of pre-existing immunity in the efficacy of HAdV-5 vector vaccines.

## Supporting information

S1 Table HAdV-5 NAb titer of the blood donors. (PDF)
