# Supplementary material for: Seroprevalence of human adenovirus type 5 neutralizing antibodies in the Philippines
Source: PLoS One. 2023 Dec 1;18(12):e0293046. doi: 10.1371/journal.pone.0293046 (PMC10691707; doi:10.1371/journal.pone.0293046)
Supplement: S1 Table — (PDF) [file pone.0293046.s001.pdf]

Supplementary Table 1. HAdV-5 NAb titer of the blood donors

	PGH SAMPLE ID NO.	HAdV-5 NAb TITER	SEROSTATUS	LEVEL OF HADV-5 TITER
1	PGHDO2021001345	15	NEGATIVE	LOW
2	PGHDO2021002157	15	NEGATIVE	LOW
3	PGHDO2021002194	256	POSITIVE	HIGH
4	PGHDO2021002298	4,096	POSITIVE	HIGH
5	PGHDO2021002419	64	POSITIVE	LOW
6	PGHDO2021002707	64	POSITIVE	LOW
7	PGHDO2021002969	15	NEGATIVE	LOW
8	PGHDO2021003096	64	POSITIVE	LOW
9	PGHDO2021003108	16	POSITIVE	LOW
10	PGHDO2021003316	64	POSITIVE	LOW
11	PGHDO2021003325	16	POSITIVE	LOW
12	PGHDO2021003327	256	POSITIVE	HIGH
13	PGHDO2021003436	64	POSITIVE	LOW
14	PGHDO2021003463	256	POSITIVE	HIGH
15	PGHDO2021003464	256	POSITIVE	HIGH
16	PGHDO2021003521	256	POSITIVE	HIGH
17	PGHDO2021003528	256	POSITIVE	HIGH
18	PGHDO2021003536	64	POSITIVE	LOW
19	PGHDO2021003538	64	POSITIVE	LOW
20	PGHDO2021003560	64	POSITIVE	LOW
21	PGHDO2021003565	256	POSITIVE	HIGH
22	PGHDO2021003676	1,024	POSITIVE	HIGH
23	PGHDO2021003787	256	POSITIVE	HIGH
24	PGHDO2021003794	256	POSITIVE	HIGH
25	PGHDO2021003796	16	POSITIVE	LOW

26	PGHDO2021003806	256	POSITIVE	HIGH
27	PGHDO2021003834	256	POSITIVE	HIGH
28	PGHDO2021003839	1,024	POSITIVE	HIGH
29	PGHDO2021003845	16	POSITIVE	LOW
30	PGHDO2021003872	256	POSITIVE	HIGH
31	PGHDO2021003905	64	POSITIVE	LOW
32	PGHDO2021003995	1,024	POSITIVE	HIGH
33	PGHDO2021004013	16	POSITIVE	LOW
34	PGHDO2021004014	256	POSITIVE	HIGH
35	PGHDO2021004019	256	POSITIVE	HIGH
36	PGHDO2021004021	15	NEGATIVE	LOW
37	PGHDO2021004024	64	POSITIVE	LOW
38	PGHDO2021004029	64	POSITIVE	LOW
39	PGHDO2021004030	1,024	POSITIVE	HIGH
40	PGHDO2021004031	15	NEGATIVE	LOW
41	PGHDO2021004032	15	NEGATIVE	LOW
42	PGHDO2021004035	256	POSITIVE	HIGH
43	PGHDO2021004039	1,024	POSITIVE	HIGH
44	PGHDO2021004040	16	POSITIVE	LOW
45	PGHDO2021004041	1,024	POSITIVE	HIGH
46	PGHDO2021004042	256	POSITIVE	HIGH
47	PGHDO2021004043	64	POSITIVE	LOW
48	PGHDO2021004045	1,024	POSITIVE	HIGH
49	PGHDO2021004047	16	POSITIVE	LOW
50	PGHDO2021004049	256	POSITIVE	HIGH
51	PGHDO2021004050	4,096	POSITIVE	HIGH
52	PGHDO2021004054	16	POSITIVE	LOW

53	PGHDO2021004055	64	POSITIVE	LOW
54	PGHDO2021004067	256	POSITIVE	HIGH
55	PGHDO2021004082	64	POSITIVE	LOW
56	PGHDO2021004090	64	POSITIVE	LOW
57	PGHDO2021004092	64	POSITIVE	LOW
58	PGHDO2021004095	15	NEGATIVE	LOW
59	PGHDO2021004096	256	POSITIVE	HIGH
60	PGHDO2021004100	1,024	POSITIVE	HIGH
61	PGHDO2021004127	15	NEGATIVE	LOW
62	PGHDO2021004160	64	POSITIVE	LOW
63	PGHDO2021004221	64	POSITIVE	LOW
64	PGHDO2021004302	15	NEGATIVE	LOW
65	PGHDO2021004352	64	POSITIVE	LOW
66	PGHDO2021004391	1,024	POSITIVE	HIGH
67	PGHDO2021004394	1,024	POSITIVE	HIGH
68	PGHDO2021004400	15	NEGATIVE	LOW
69	PGHDO2021004406	256	POSITIVE	HIGH
70	PGHDO2021004407	64	POSITIVE	LOW
71	PGHDO2021004408	256	POSITIVE	HIGH
72	PGHDO2021004620	64	POSITIVE	LOW
73	PGHDO2021004675	16	POSITIVE	LOW
74	PGHDO2021004692	15	NEGATIVE	LOW
75	PGHDO2021004739	256	POSITIVE	HIGH
76	PGHDO2021004745	16,384	POSITIVE	HIGH
77	PGHDO2021004783	15	NEGATIVE	LOW

78	PGHDO2021004792	64	POSITIVE	LOW
79	PGHDO2021004819	4,096	POSITIVE	HIGH
80	PGHDO2021004830	1,024	POSITIVE	HIGH
81	PGHDO2021004836	64	POSITIVE	LOW
82	PGHDO2021004853	256	POSITIVE	HIGH
83	PGHDO2021004867	256	POSITIVE	HIGH
84	PGHDO2021004869	1,024	POSITIVE	HIGH
85	PGHDO2021004874	16,384	POSITIVE	HIGH
86	PGHDO2021004881	1,024	POSITIVE	HIGH
87	PGHDO2021004885	256	POSITIVE	HIGH
88	PGHDO2021004890	1,024	POSITIVE	HIGH
89	PGHDO2021004903	4,096	POSITIVE	HIGH
90	PGHDO2021004904	256	POSITIVE	HIGH
91	PGHDO2021004914	16	POSITIVE	LOW
92	PGHDO2021004917	256	POSITIVE	HIGH
93	PGHDO2021004924	256	POSITIVE	HIGH
94	PGHDO2021004979	64	POSITIVE	LOW
95	PGHDO2021004990	1,024	POSITIVE	HIGH
96	PGHDO2021005028	1,024	POSITIVE	HIGH
97	PGHDO2021005029	1,024	POSITIVE	HIGH
98	PGHDO2021005033	15	NEGATIVE	LOW
99	PGHDO2021005034	256	POSITIVE	HIGH
100	PGHDO2021005055	16	POSITIVE	LOW
101	PGHDO2021005111	1,024	POSITIVE	HIGH
102	PGHDO2021005130	256	POSITIVE	HIGH
103	PGHDO2021005136	1,024	POSITIVE	HIGH
104	PGHDO2021005148	15	NEGATIVE	LOW
105	PGHDO2021005181	16	POSITIVE	LOW
106	PGHDO2021005244	256	POSITIVE	HIGH

107	PGHDO2021005245	16	POSITIVE	LOW
108	PGHDO2021005286	16	POSITIVE	LOW
109	PGHDO2021005493	64	POSITIVE	LOW
110	PGHDO2021005596	256	POSITIVE	HIGH
111	PGHDO2021005600	15	NEGATIVE	LOW
112	PGHDO2021005602	256	POSITIVE	HIGH
113	PGHDO2021005608	15	NEGATIVE	LOW
114	PGHDO2021005617	16	POSITIVE	LOW
115	PGHDO2021005624	64	POSITIVE	LOW
116	PGHDO2021005627	64	POSITIVE	LOW
117	PGHDO2021005642	1,024	POSITIVE	HIGH
118	PGHDO2021005660	64	POSITIVE	LOW
119	PGHDO2021005661	256	POSITIVE	HIGH
120	PGHDO2021005676	64	POSITIVE	LOW
121	PGHDO2021005690	64	POSITIVE	LOW
122	PGHDO2021005697	16	POSITIVE	LOW
123	PGHDO2021005707	256	POSITIVE	HIGH
124	PGHDO2021005711	64	POSITIVE	LOW
125	PGHDO2021005712	256	POSITIVE	HIGH
126	PGHDO2021005713	256	POSITIVE	HIGH
127	PGHDO2021005718	16	POSITIVE	LOW
128	PGHDO2021005720	64	POSITIVE	LOW
129	PGHDO2021005721	256	POSITIVE	HIGH
130	PGHDO2021005728	15	NEGATIVE	LOW
131	PGHDO2021005729	1,024	POSITIVE	HIGH
132	PGHDO2021006086	64	POSITIVE	LOW
133	PGHDO2021006107	64	POSITIVE	LOW
134	PGHDO2021006113	4,096	POSITIVE	HIGH

135	PGHDO2021006129	64	POSITIVE	LOW
136	PGHDO2021006159	1,024	POSITIVE	HIGH
137	PGHDO2021006161	256	POSITIVE	HIGH
138	PGHDO2021006164	15	NEGATIVE	LOW
139	PGHDO2021006220	64	POSITIVE	LOW
140	PGHDO2021006239	256	POSITIVE	HIGH
141	PGHDO2021006245	64	POSITIVE	LOW
142	PGHDO2021006271	16	POSITIVE	LOW
143	PGHDO2021006761	64	POSITIVE	LOW
144	PGHDO2021006779	15	NEGATIVE	LOW
145	PGHDO2021006787	1,024	POSITIVE	HIGH
146	PGHDO2021006797	256	POSITIVE	HIGH
147	PGHDO2021006828	15	NEGATIVE	LOW
148	PGHDO2021006831	16	POSITIVE	LOW
149	PGHDO2021006880	64	POSITIVE	LOW
150	PGHDO2021006888	64	POSITIVE	LOW
151	PGHDO2021006901	256	POSITIVE	HIGH
152	PGHDO2021006904	15	NEGATIVE	LOW
153	PGHDO2021006913	15	NEGATIVE	LOW
154	PGHDO2021006917	16	POSITIVE	LOW
155	PGHDO2021006922	64	POSITIVE	LOW
156	PGHDO2021006929	64	POSITIVE	LOW
157	PGHDO2021006950	256	POSITIVE	HIGH
158	PGHDO2021007072	256	POSITIVE	HIGH
159	PGHDO2021007149	64	POSITIVE	LOW
160	PGHDO2021007153	256	POSITIVE	HIGH
161	PGHDO2021007159	256	POSITIVE	HIGH
162	PGHDO2021007169	1,024	POSITIVE	HIGH
163	PGHDO2021007172	256	POSITIVE	HIGH
164	PGHDO2021007214	1,024	POSITIVE	HIGH
165	PGHDO2021007599	256	POSITIVE	HIGH

166	PGHDO2021007600	15	NEGATIVE	LOW
167	PGHDO2021007605	64	POSITIVE	LOW
168	PGHDO2021007606	16	POSITIVE	LOW
169	PGHDO2021007724	1,024	POSITIVE	HIGH
170	PGHDO2021007727	1,024	POSITIVE	HIGH
171	PGHDO2021007730	64	POSITIVE	LOW
172	PGHDO2021007737	256	POSITIVE	HIGH
173	PGHDO2021007739	64	POSITIVE	LOW
174	PGHDO2021007740	256	POSITIVE	HIGH
175	PGHDO2021007741	1,024	POSITIVE	HIGH
176	PGHDO2021007742	1,024	POSITIVE	HIGH
177	PGHDO2021007743	15	NEGATIVE	LOW
178	PGHDO2021007745	256	POSITIVE	HIGH
179	PGHDO2021007747	1,024	POSITIVE	HIGH
180	PGHDO2021007755	256	POSITIVE	HIGH
181	PGHDO2021007848	1,024	POSITIVE	HIGH
182	PGHDO2021007854	1,024	POSITIVE	HIGH
183	PGHDO2021007938	256	POSITIVE	HIGH
184	PGHDO2021007941	1,024	POSITIVE	HIGH
185	PGHDO2021007943	1,024	POSITIVE	HIGH
186	PGHDO2021008011	64	POSITIVE	LOW
187	PGHDO2021008284	256	POSITIVE	HIGH
188	PGHDO2021008304	64	POSITIVE	LOW
189	PGHDO2021008306	256	POSITIVE	HIGH
190	PGHDO2021008308	1,024	POSITIVE	HIGH
191	PGHDO2021008309	1,024	POSITIVE	HIGH
192	PGHDO2021008310	4,096	POSITIVE	HIGH
193	PGHDO2021008311	1,024	POSITIVE	HIGH
194	PGHDO2021008312	256	POSITIVE	HIGH
195	PGHDO2021008313	16,385	POSITIVE	HIGH
196	PGHDO2021008314	64	POSITIVE	LOW

197	PGHDO2021008315	4,096	POSITIVE	HIGH
198	PGHDO2021008317	256	POSITIVE	HIGH
199	PGHDO2021008318	256	POSITIVE	HIGH
200	PGHDO2021008319	1,024	POSITIVE	HIGH
201	PGHDO2021008320	256	POSITIVE	HIGH
202	PGHDO2021008321	256	POSITIVE	HIGH
203	PGHDO2021008322	16	POSITIVE	LOW
204	PGHDO2021008323	1,024	POSITIVE	HIGH
205	PGHDO2021008325	256	POSITIVE	HIGH
206	PGHDO2021008326	1,024	POSITIVE	HIGH
207	PGHDO2021008327	4,096	POSITIVE	HIGH
208	PGHDO2021008328	64	POSITIVE	LOW
209	PGHDO2021008331	64	POSITIVE	LOW
210	PGHDO2021008332	64	POSITIVE	LOW
211	PGHDO2021008333	256	POSITIVE	HIGH
212	PGHDO2021008335	256	POSITIVE	HIGH
213	PGHDO2021008336	256	POSITIVE	HIGH
214	PGHDO2021008337	256	POSITIVE	HIGH
215	PGHDO2021008338	1,024	POSITIVE	HIGH
216	PGHDO2021008339	16,385	POSITIVE	HIGH
217	PGHDO2021008342	16	POSITIVE	LOW
218	PGHDO2021008343	64	POSITIVE	LOW
219	PGHDO2021008344	1,024	POSITIVE	HIGH
220	PGHDO2021008345	4.096	POSITIVE	HIGH
221	PGHDO2021008346	256	POSITIVE	HIGH
222	PGHDO2021008347	256	POSITIVE	HIGH
223	PGHDO2021008601	15	NEGATIVE	LOW
224	PGHDO2021008608	16	POSITIVE	LOW
225	PGHDO2021008877	256	POSITIVE	HIGH
226	PGHDO2021009071	15	NEGATIVE	LOW
227	PGHDO2021009072	15	NEGATIVE	LOW

228	PGHDO2021009073	15	NEGATIVE	LOW
229	PGHDO2021009076	64	POSITIVE	LOW
230	PGHDO2021009079	256	POSITIVE	HIGH
231	PGHDO2021009081	1,024	POSITIVE	HIGH
232	PGHDO2021009082	15	NEGATIVE	LOW
233	PGHDO2021009085	1,024	POSITIVE	HIGH
234	PGHDO2021009088	64	POSITIVE	LOW
235	PGHDO2021009090	15	NEGATIVE	LOW
236	PGHDO2021009111	64	POSITIVE	LOW
237	PGHDO2021009547	64	POSITIVE	LOW
238	PGHDO2021010963	64	POSITIVE	LOW
239	PGHDO2021010981	15	NEGATIVE	LOW
240	PGHDO2021010985	64	POSITIVE	LOW
241	PGHDO2021010998	256	POSITIVE	HIGH
242	PGHDO2021011029	256	POSITIVE	HIGH
243	PGHDO2021011032	1,024	POSITIVE	HIGH
244	PGHDO2021011037	64	POSITIVE	LOW
245	PGHDO2021011043	15	NEGATIVE	LOW
246	PGHDO2021011072	256	POSITIVE	HIGH
247	PGHDO2021011087	16	POSITIVE	LOW
248	PGHDO2021011200	256	POSITIVE	HIGH
249	PGHDO2021011209	15	NEGATIVE	LOW

250	PGHDO2021011233	256	POSITIVE	HIGH
251	PGHDO2021011238	256	POSITIVE	HIGH
252	PGHDO2021011289	1,024	POSITIVE	HIGH
253	PGHDO2021011320	256	POSITIVE	HIGH
254	PGHDO2021011655	16	POSITIVE	LOW
255	PGHDO2021011660	15	NEGATIVE	LOW
256	PGHDO2021011714	15	NEGATIVE	LOW
257	PGHDO2021011865	256	POSITIVE	HIGH
258	PGHDO2021011901	64	POSITIVE	LOW
259	PGHDO2021012024	64	POSITIVE	LOW
260	PGHDO2021012042	256	POSITIVE	HIGH
261	PGHDO2021012056	256	POSITIVE	HIGH
262	PGHDO2021012064	256	POSITIVE	HIGH
263	PGHDO2021012256	256	POSITIVE	HIGH
264	PGHDO2021012259	64	POSITIVE	LOW
265	PGHDO2021012452	1,024	POSITIVE	HIGH
266	PGHDO2021012460	15	NEGATIVE	LOW
267	PGHDO2021012470	1,024	POSITIVE	HIGH
268	PGHDO2021012580	15	NEGATIVE	LOW
269	PGHDO2021012620	256	POSITIVE	HIGH
270	PGHDO2021012688	16	POSITIVE	LOW
271	PGHDO2021012767	256	POSITIVE	HIGH
272	PGHDO2021012778	256	POSITIVE	HIGH
273	PGHDO2021012797	64	POSITIVE	LOW
274	PGHDO2021012799	64	POSITIVE	LOW
275	PGHDO2021012800	64	POSITIVE	LOW

276	PGHDO2021012827	1,024	POSITIVE	HIGH
277	PGHDO2021012844	1,024	POSITIVE	HIGH
278	PGHDO2021012845	1,024	POSITIVE	HIGH
279	PGHDO2021012859	16	POSITIVE	LOW
280	PGHDO2021012860	64	POSITIVE	LOW
281	PGHDO2021012900	256	POSITIVE	HIGH
282	PGHDO2021012915	256	POSITIVE	HIGH
283	PGHDO2021013031	15	NEGATIVE	LOW
284	PGHDO2021013052	64	POSITIVE	LOW
285	PGHDO2021013190	256	POSITIVE	HIGH
286	PGHDO2021013195	64	POSITIVE	LOW
287	PGHDO2021013603	1,024	POSITIVE	HIGH
288	PGHDO2021013672	15	NEGATIVE	LOW
289	PGHDO2021013918	1,024	POSITIVE	HIGH
290	AB3302	1,024	POSITIVE	HIGH
291	AB1205	256	POSITIVE	HIGH
292	AA5901	256	POSITIVE	HIGH
293	AA6401	256	POSITIVE	HIGH
294	AB1301	256	POSITIVE	HIGH
295	AB1401	256	POSITIVE	HIGH
296	AB5301	64	POSITIVE	LOW
297	AA1002	16,385	POSITIVE	HIGH
298	AA6001	16	POSITIVE	LOW
299	AB1701	256	POSITIVE	HIGH
300	AB1801	256	POSITIVE	HIGH
301	AB1204	256	POSITIVE	HIGH
302	AB4101	256	POSITIVE	HIGH
303	AB5001	16	POSITIVE	LOW
304	AB3301	16	POSITIVE	LOW
305	AB0201	64	POSITIVE	LOW
306	AB1208	15	NEGATIVE	LOW

307	AB0402	16	POSITIVE	LOW
308	AB0301	256	POSITIVE	HIGH
309	AB1101	1,024	POSITIVE	HIGH
310	AB3601	16,385	POSITIVE	HIGH
311	AA7604	1,024	POSITIVE	HIGH
312	AA7606	1,024	POSITIVE	HIGH
313	AA6701	64	POSITIVE	LOW
314	AB5401	256	POSITIVE	HIGH
315	AA7702	16,385	POSITIVE	HIGH
316	AA5203	64	POSITIVE	LOW
317	AA5206	64	POSITIVE	LOW
318	AA5303	256	POSITIVE	HIGH
319	AA2801	256	POSITIVE	HIGH
320	AB0801	64	POSITIVE	LOW
321	AB0401	16	POSITIVE	LOW
322	AB1206	15	NEGATIVE	LOW
323	AB3903	15	NEGATIVE	LOW
324	AA7101	16	POSITIVE	LOW
325	AA2201	256	POSITIVE	HIGH
326	AA2101	1,024	POSITIVE	HIGH
327	AA1901	256	POSITIVE	HIGH
328	AA2401	1,024	POSITIVE	HIGH
329	AA0801	256	POSITIVE	HIGH
330	AA2001	256	POSITIVE	HIGH
331	AA2301	1,024	POSITIVE	HIGH
332	AA5202	1,024	POSITIVE	HIGH
333	AA5401	256	POSITIVE	HIGH
334	AA1101	16,385	POSITIVE	HIGH
335	AA2501	64	POSITIVE	LOW
336	AA1301	256	POSITIVE	HIGH
337	AA1502	15	NEGATIVE	LOW

338	AA2102	256	POSITIVE	HIGH
339	AA2003	4,096	POSITIVE	HIGH
340	AA0301	64	POSITIVE	LOW
341	AA2402	1,024	POSITIVE	HIGH
342	AA2002	256	POSITIVE	HIGH
343	AA5102	64	POSITIVE	LOW
344	AA1203	256	POSITIVE	HIGH
345	AA0401	1,024	POSITIVE	HIGH
346	AA0402	256	POSITIVE	HIGH
347	AA6301	256	POSITIVE	HIGH
348	AB0901	15	NEGATIVE	LOW
349	AA6601	16	POSITIVE	LOW
350	AA0501	64	POSITIVE	LOW
351	AA1302	1,024	POSITIVE	HIGH
352	AA0201	64	POSITIVE	LOW
353	AA2701	64	POSITIVE	LOW
354	AA0103	16,385	POSITIVE	HIGH
355	AC2201	1,024	POSITIVE	HIGH
356	AC2602	64	POSITIVE	LOW
357	AC0502	1,024	POSITIVE	HIGH
358	AC6201	64	POSITIVE	LOW
359	AC6301	4,096	POSITIVE	HIGH
360	AC2901	1,024	POSITIVE	HIGH
361	AC3101	15	NEGATIVE	LOW
362	AC1601	16	POSITIVE	LOW
363	AC1201	1,024	POSITIVE	HIGH
364	AC3201	1,024	POSITIVE	HIGH
365	AC2101	256	POSITIVE	HIGH
366	AC0503	16	POSITIVE	LOW
367	AC1602	1,024	POSITIVE	HIGH
368	AA0803	1,024	POSITIVE	HIGH

369	AA1601	1,024	POSITIVE	HIGH
370	AA7001	16,385	POSITIVE	HIGH
371	AB3402	64	POSITIVE	LOW
372	AB3004	64	POSITIVE	LOW
373	AB0605	1,024	POSITIVE	HIGH
374	AB4601	256	POSITIVE	HIGH
375	AB6202	16	POSITIVE	LOW
376	AB4901	256	POSITIVE	HIGH
377	AB0603	1,024	POSITIVE	HIGH
378	AB6405	16,385	POSITIVE	HIGH
379	AB3201	1,024	POSITIVE	HIGH
380	AB2001	16	POSITIVE	LOW
381	AB4701	256	POSITIVE	HIGH
382	AB6301	256	POSITIVE	HIGH
383	AB3904	1,024	POSITIVE	HIGH
384	AB6701	16,385	POSITIVE	HIGH
385	AB6201	256	POSITIVE	HIGH
386	AB8001	256	POSITIVE	HIGH
387	AB6602	1,024	POSITIVE	HIGH
388	AB5902	1,024	POSITIVE	HIGH
389	AB3501	4,096	POSITIVE	HIGH
390	AB6102	256	POSITIVE	HIGH
391	AB6501	4,096	POSITIVE	HIGH
